# A Global Research Agenda for Pediatric HIV

**DOI:** 10.1097/QAI.0000000000001743

**Published:** 2018-07-11

**Authors:** Martina Penazzato, Cadi Irvine, Marissa Vicari, Shaffiq M. Essajee, Aditi Sharma, Thanyawee Puthanakit, Elaine J. Abrams, Meg Doherty

**Affiliations:** * Department of HIV and Global Hepatitis Programme, World Health Organization, Geneva, Switzerland;; † Department of HIV Programmes and Advocacy, International AIDS Society, Geneva, Switzerland;; ‡ HIV Section, Program Division, UNICEF, New York, NY;; § Global Network of People Living with HIV (GNP+);; ║ Department of Pediatrics, Center of Excellence in Pediatric Infectious Diseases and Vaccine, Faculty of Medicine, Chulalongkorn University; and; ¶ ICAP at Columbia University, Mailman School of Public Health, and College of Physicians & Surgeons, Columbia University, New York, NY.

**Keywords:** priorities, children, HIV, antiretrovirals, HIV diagnosis, service delivery

## Abstract

**Background:**

Despite progress, 2016 still saw 160,000 new infections and 120,000 AIDS-related deaths among children. Evidence gaps on how to best diagnose, treat, and deliver services to children living with HIV remain. A global research prioritization exercise was undertaken by WHO and CIPHER to focus research efforts in the context of diminishing resources.

**Methods:**

The Child Health and Nutrition Research Initiative methodology was adapted and used, as described by Irvine et al.^1^ Outcomes were reviewed by an expert group and 5 priority themes identified for testing, antiretroviral treatment, and service delivery, accounting for existing policies, published literature and ongoing research.

**Results:**

A total of 749 questions were submitted by 269 individuals from 62 countries. For HIV testing, priority themes included strategies and interventions to improve access, uptake and linkage to care, including with novel diagnostic tools and entry points beyond antenatal care. For treatment, priorities included strategies to improve adherence, short- and long-term outcomes and management of coinfections, optimal drug formulations, and early ART. For service delivery, priorities included strategies or interventions to improve access, uptake and retention in care, including psychosocial and family support and approaches to HIV disclosure and reduction of stigma and discrimination.

**Conclusions:**

This is the largest Child Health and Nutrition Research Initiative exercise undertaken in HIV. The results provide guidance to focus future research in pediatric HIV for impact. Global commitment to support priority research, adequate investment, and strong leadership is urgently needed to improve the health and well-being of children living with and affected by HIV.

## Abstract 2

Supplemental Digital Content is Available in the Text.

## INTRODUCTION

An estimated 2.1 million (1.7–2.6 million) children were living with HIV globally in 2016, with less than 50% of these children receiving antiretroviral (ARV) treatment.^2^ Despite steady decreases, 2016 still saw 160,000 new infections and 120,000 AIDS-related deaths among children.^3^ A number of global initiatives aim at closing the treatment gap for pediatric HIV and the THREE FREES framework provides the platform to reach “super-fast track targets” and end the AIDS epidemic among children by 2020 with ambitious treatment goals by 2018.^4,5^

Critical barriers to scaling up pediatric HIV treatment and care remain, including the complexity of existing approaches to testing and treating children, lack of age-appropriate formulations to provide the most effective and tolerable drugs, and lingering difficulties around decentralizing and integrating HIV services into the broader maternal and child health platform.^5^ Innovative testing, ARV treatment, and service delivery strategies are needed to simplify and expand pediatric services, but evidence to support this is currently lacking. Updated and robust research is required to accelerate the introduction of innovations, overcome existing implementation challenges, and inform the development of normative guidance that will set the standard of care for children living with HIV around the world. To overcome these challenges in the context of increasing funding constraints, efforts must be focused on generating targeted evidence that improves HIV program implementation through a better understanding of what works for infants and children.

To this end, the World Health Organization (WHO) and the Collaborative Initiative for Paediatric HIV Education and Research (CIPHER) of the International AIDS Society (IAS) undertook a global research prioritization process with broad engagement of stakeholders. The approach sought input into 3 research themes: testing, ARV treatment, and service delivery with a view to informing global policy change, and improving outcomes for infants and children living with and affected by HIV.

## METHODS

The Child Health and Nutrition Research Initiative (CHNRI) methodology,^7,8^ a well-established approach for setting health research priorities, was adapted and used for this process. For more details on the different steps of the process, see full methods by Irvine and colleagues.^1^

In brief, 5 phases were carried out to set research priorities in pediatric HIV, as follows. First, an expert working group was convened to define the scope of the exercise. In phase 2, an online survey was disseminated widely to collect priority research questions. Respondents were asked to tag their questions within the relevant research area (testing, treatment, or service delivery) and the research domain (descriptive discovery, development, and delivery—Table 1). Phase 3 consisted of thematic coding, analysis, and consolidation of the questions submitted to form a collated list. In phase 4, the collated list was sent in a second survey to respondents of the first survey for scoring against predefined criteria (answerability, impact, implementation, and equity). The total research priority score (RPS) and the Average Expert Agreement (AEA) scores were calculated to obtain the final ranking.^9^ Finally, in phase 5, the outcome of the CHNRI process was then reviewed by selected pediatric HIV experts charged to identify the top 5 priority themes among the top 10 ranked research questions in each topic area (testing, treatment, and service delivery) accounting for existing policies, systematic reviews, published literature, and planned and ongoing research. This last phase was a novel addition to the established CHNRI method to better communicate and contextualize the final themes within the current research landscape.

**TABLE 1.**
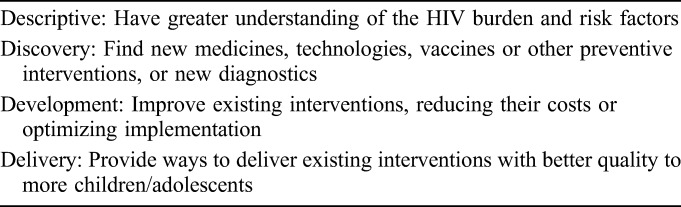
Research Question Domain Type

## RESULTS

Overall, an average of 2.8 pediatric HIV research questions per person were submitted (total of 749 research questions) by 269 individuals from 62 countries, across all WHO regions. After thematic content analysis, the final collated lists included 51 questions covering testing, ARV treatment, or service delivery.

Eighty-three respondents scored a total of 16 HIV testing research questions. For the top 10 ranked questions the overall mean RPS was 84 [range 79.8 – 89.4] and the mean AEA was 70 [range 61 – 79]. Among these, the types of research that were ranked most frequently among the top 10 were delivery (n = 5) and development (n = 3) questions, whereas fewer discovery (n = 1) and descriptive (n = 1) research questions made the top 10.

A total of 21 treatment research questions were scored by respondents (n = 66). For the top 10 ranked questions, the overall mean RPS was 83 (78.2–87.6) and the mean AEA was 68 (61–75). Among the top 10 ranked research ideas, discovery (n = 4) and development (n = 3) were ranked more highly than descriptive (n = 2) and delivery (n = 1) type research questions.

For service delivery, a total of 14 research questions were scored by respondents (n = 62). The mean RPS for the top 10 questions was 84 (79.8–89.4) and the mean AEA was 70 (61–79). Among the top 10 ranked research ideas, development (n = 6) was ranked more highly than descriptive (n = 2) and delivery (n = 2) type research questions with no discovery featured in the top 10.

Generally, the questions with the greatest level of overall agreement also achieved higher overall RPSs. The full lists of questions per research area are provided in Table 1, Supplemental Digital Content, http://links.lww.com/QAI/B167. The top 5 priority themes that were elaborated by the expert group are shown in Table 2. The final 5 themes identified in each area by the end of the exercise address the following: for HIV testing, priority themes included strategies and interventions to improve access, uptake and linkage to care, optimal placement and timing of novel diagnostic tools for point-of-care (POC) use and effective testing strategies at entry points other than antenatal care (ANC) for identifying undiagnosed HIV-positive infants and children in different epidemic settings; for ARV treatment, priorities included strategies to monitor and improve adherence, prevent and manage coinfections, short- and long-term outcomes (particularly in the context of very early ART) and safety, efficacy, acceptability, pharmacokinetics of existing and new ARV drugs and formulations; for service delivery, priorities included service delivery models across the cascade, strategies or interventions to improve access to, uptake of and retention in care, to reduce stigma and discrimination experienced by children and their caregivers and the provision of psychosocial and family support, particularly around disclosure.

**TABLE 2.**
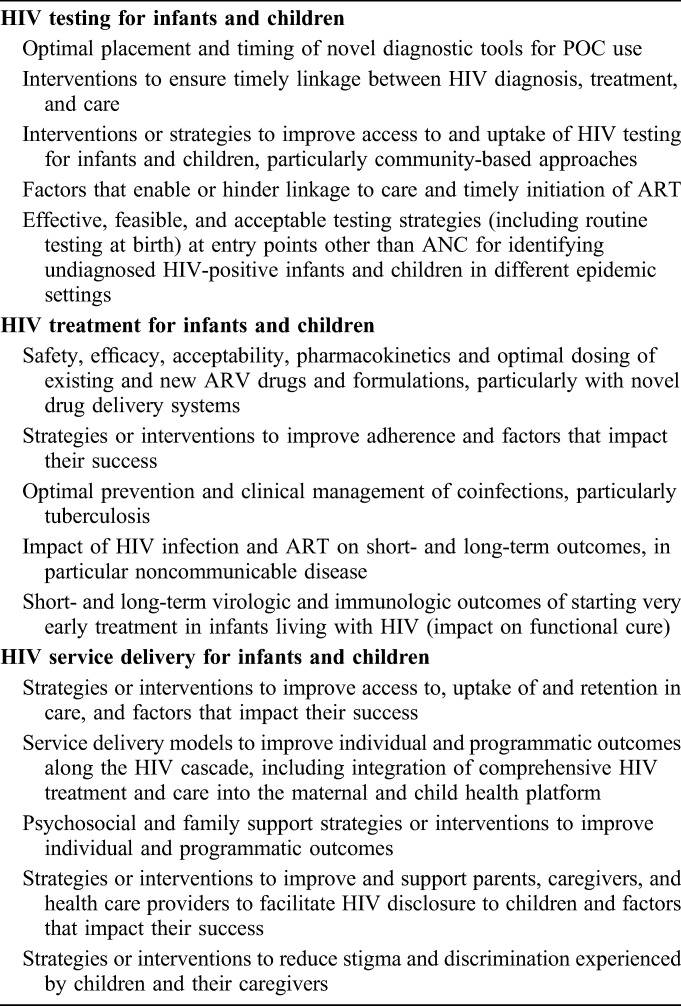
Top 5 Priority Themes for Pediatric HIV Testing, Treatment, and Service Delivery

## DISCUSSION

To our knowledge, this is the largest research prioritization exercise undertaken in the field of pediatric HIV. The process reflects the thinking of a broad range of stakeholders and overall highlights the need for more operational research to be conducted in the field of pediatric HIV. It should be recognized that the outcome of this process is more likely to reflect the context in Sub-Saharan Africa, as this is where 80% of the global pediatric HIV burden is, and where most of the respondents belonged. Below, we reflect on the outcomes of the exercise for the 3 key areas and on how the prioritized themes should be seen in the context of our current knowledge and ongoing research.

### HIV Testing

Overall, the ranking of questions for HIV testing suggests that although valuable tools and strategies are at our disposal, what is really needed is improved knowledge on how to combine and target key tools and interventions to maximize their impact. Uptake of and access to pediatric HIV testing are highly variable across settings with only 43% of HIV-exposed infants tested by the recommended age of 2 months.^1^ There is a lack of evidence for HIV testing and counseling approaches that work for children, especially outside the traditional entry points of ANC and maternity wards.^9,10^ Overlooked entry points such as malnutrition units, tuberculosis units, immunization clinics, and inpatient wards may provide a more efficient way to identify HIV-infected children and link them to treatment and care.^11–13^ Community-based testing approaches (ie, home-based, family-centered and outreach) for this age group have also shown promise and acceptability but require further defining, with data on feasibility and cost-effectiveness before countries scale-up specific strategies.^13^ Development of novel POC tools for infant diagnosis of HIV, including testing at birth, is underway and 2 POC EID technologies received WHO prequalification in 2016.^14^ These technologies have quicker turnaround times for test results and increased ART initiation rates compared with conventional laboratory systems.^15–17^ However, where and when to use these diagnostic tools needs to be further defined to optimize implementation efforts. Evaluating the impact that introducing POC EID has on key outcomes including ART initiation rates and mortality reduction will also be important going forward.

Once tested for HIV, ensuring timely linkage to treatment and care is critical. Only 43% (30–54) of all children living with HIV were accessing ARV treatment in 2016.^3^ Determining what factors ensure that children living with HIV receive lifesaving treatment in a timely manner will help guide effective strategies. In 2017, WHO recommended rapid initiation of ART, based on encouraging results in adult studies with improved program outcomes, including reduced loss to follow-up in the pre-ART period.^18^ The recommendation also applies for children; although it should be noted that the evidence for rapid initiation of ART in children is weaker than for adults with research indicating that for sick children, immediate ART may not lead to better outcomes than starting ART after initial stabilization in the hospital.^19–21^ Although ART initiation remains a priority for this age group, especially for children younger than 5 years and children who present with symptoms, timely provision of appropriate care for clinical conditions requiring acute management is the first priority.

There are studies in the pipeline that promise to shine light on and provide practical solutions to some of the priority thematic areas discussed above, including: testing at family planning clinics^22^; targeted provider-initiated testing and counselling^23^; uptake of POC HIV testing^24^; impact of ARVs exposure in HIV-exposed infants and young children; and impact of financial incentives to increase pediatric HIV testing.^25^

### HIV Treatment

Pediatric HIV treatment has come a long way since the beginning of the epidemic but the general lack of well-tolerated age-appropriate formulations remains a key barrier to effective scale-up. ARV options remain limited with suboptimal regimens still being used in more than half of children on ARV treatment^26^ with clear implications on virological suppression rate and selection of HIV drug resistance.^4^ This is particularly important in the context of very early ART for neonates and infants, which holds promise for achieving functional cure in the future. The third pediatric conference on ARV drug optimization took place in 2016 to take stock of progress made and reflect on remaining challenges.^27^ The outcomes of this consultation highlighted the evidence gaps to be addressed to further advance the pediatric treatment optimization agenda, which were largely confirmed during this larger research prioritization process.^28^ New child-friendly FDCs of the currently WHO recommended regimen are still being tested and while some are in advanced stage of development, a better understanding of the acceptability and operational issues related to their introduction and use is still required.^29^ New formulations of dolutegravir have recently received FDA approval and trials are investigating its use in lower weight bands and ages, but limited data are available to inform subsequent sequencing once dolutegravir has been used.^30^ Evidence to inform the use of TAF has been long awaited with approval for use in children expected for late 2018 or early 2019.^31^ There are also promising long-acting drug delivery systems in the pipeline, which are attractive in their potential to overcome poor adherence but problematic when dealing with a growing body and changing pharmacokinetics.^32^ It is clear that further evidence is still needed on the safety, efficacy, acceptability, pharmacokinetics, and optimal dosing of existing and new ARV drugs and formulations, particularly with upcoming novel drugs and delivery systems.

It is increasingly recognized that HIV infection and/or exposure to ARVs during the critical developmental stage of early childhood may have a short- and long-term impact on health with increased risk for a range of noncommunicable diseases (NCDs). This growing field of research is important to ensure that children not only survive but also thrive and transform into healthy adolescents and adults. Identifying the most common comorbidities, particularly noncommunicable diseases, to consider for this population with timely and effective screening and appropriate interventions will be key. How the timing of ART initiation affects virological suppression and these broader outcomes in the long run deserves well-planned research that also accounts for the epidemiology of these diseases in the regions of the world where HIV is most prevalent.

Infants and children face the longest battle to remain adherent to ARVs and little evidence exists on the best approaches to facilitate this across their lifetime. Effective strategies or interventions that can support and simplify ARV adherence for children and importantly for their caregivers are urgently needed. WHO recommends a range of behavioral and biomedical interventions that have been shown to be successful including use of peer counselors, mobile phone text messages, reminder devices, cognitive-behavioral therapy, behavioral skills training, medication adherence training, fixed-dose combinations, and once-daily regimens.^18^ Ensuring that these or other effective strategies cater to the needs of caregivers and older children is critical to ensure improved adherence and improved clinical outcomes.

In the context of comorbidities where HIV is highly prevalent, attention should be given to coinfections. AIDS-related infections (particularly tuberculosis) and bacterial infections continue to be leading causes of hospital admission and mortality in adults and children living with HIV worldwide.^33^ One million children (0–14 years of age) fell ill with TB, and 250,000 children (including children with HIV) died from the disease in 2016. Tuberculosis is particularly difficult to diagnose in children and more so in HIV-infected children.^34^ Efforts to prevent TB remain unsuccessful in many countries and better strategies to provide INH preventive therapy, to best use diagnostic tools available, and to simplify TB-HIV cotreatment with tolerable regimens in the minimal number of pills are urgently needed. These needs are not unique to tuberculosis and should be considered for other coinfections such as hepatitis B or C.

### HIV Service Delivery

Under the broad umbrella of service delivery, a number of areas have emerged as items of critical importance for future research. These areas reflect a greater attention to the overall well-being of the child and the family with the goal of providing comprehensive care that maximizes survival and improves quality of life.

In this context, there is a need to identify optimal strategies to integrate multiple services with the goal of expanding access, increasing uptake, and ensuring retention in care. Although the notion of integration of HIV services into the maternal newborn and child health (MNCH) platform is not new, this has not been widely implemented because of several competing demands and lack of human resources to address all needs.^35^ Compelling models that improve individual and programmatic outcomes along the HIV cascade and that are cost-effective and feasible in different settings need to be investigated to support advocacy and rational joint programming at country level. This will entail reflecting on how to best support task shifting and decentralization with appropriate capacity building.

Living with HIV brings many challenges that cannot be managed by biomedical interventions alone. These challenges have a major impact on the well-being of the child and their carers. These include, but are not limited to, the complex matter of disclosing their HIV status to the child, reducing stigma and discrimination experienced by the child and the family, and ensuring sufficient psychosocial support to promote the child's healthy development and well-being. Disclosure of HIV status emerged as a strong theme in the research prioritization exercise and strategies or interventions to improve and support parents, caregivers, and health care providers to facilitate HIV disclosure to children was clearly prioritized. Disclosure decisions are complex because of stigma, social support concerns, family relations, parenting skills, and concern about a child's emotional and cognitive ability to understand and cope with the nature of the illness.^36^ Despite these complexities, lack of disclosure ultimately adversely affects the well-being of the child, including the uptake, adherence and retention in pediatric HIV treatment and care and needs to be addressed through better policies, appropriate HCW training and innovative strategies to account for cultural and social background.

### Considerations for Implementing the Research Agenda

As the pediatric community moves toward addressing some of the priority themes identified, acknowledgement should be made of a number of considerations that affect the way we undertake studies and the ability to generalize them more broadly. Table 3 provides suggested actions to support the adoption of the research agenda.

**TABLE 3.**
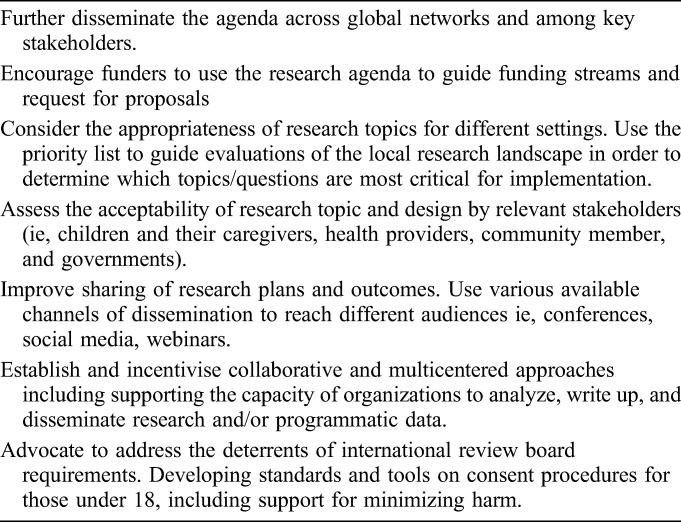
Suggested Stakeholder Actions to Support implementation of the Prioritized Research Agenda

### Limitations

Many of the limitations within this research prioritization exercise are intrinsic to the CHNRI methodology, as described in greater detail in Irvine et al.^1^ The identification of the priority themes in phase 5, which were developed in consideration of the current pediatric HIV research context, may have led to the omission of specific nuances of the research questions. To help address this, the full list of prioritized research questions is available here as separate a supplementary table, and the authors encourage accessing this information when implementing the research agendas because it provides further granularity and may be helpful in adapting the questions to different contexts. One main strength of this particular exercise lies in the contribution of a large number of research questions from a broad cross-section of geographically diverse and multidisciplinary stakeholders. Finally, it is important to note that the exercise was focused on pediatric HIV and while we recognize the critical importance of the agenda to prevent new infections, this was beyond the scope of this work.

## CONCLUSIONS

Far too many children are still acquiring HIV and dying of AIDS-related illnesses. The pediatric HIV response requires urgent acceleration and a long-term vision with evidence-based interventions to maximize short- and long-term outcomes, at both individual and programmatic levels. The range of research themes provided here is reflective of the knowledge gaps remaining for infants and children. It is important that the tremendous efforts and investments that have been made over the last decades are not wasted by failing to address the final gaps in the HIV response for these populations. To ensure that, the outcomes of this process result in high-quality research with the greatest impact in the priority thematic areas identified require greater commitment, collaboration, investment, and leadership. New research addressing the priorities identified in this process will be essential for reaching global targets for an AIDS FREE generation by 2030.

## References

[1] Irvine C Armstrong A Nagata JM Setting global research priorities in pediatric and adolescent HIV using the Child Health and Nutrition Research Initiative (CHNRI) methodology. J Acquir Immune Defic Syndr. 2018;78(suppl 1):S3–S9. 29994913 10.1097/QAI.0000000000001742 PMC6075898

[2] UNICEF, UNAIDS and WHO. Global AIDS monitoring data. 2017 Available at: http://www.unaids.org/en/resources/fact-sheet.

[3] UNAIDS. AIDSinfo. Available at: http://aidsinfo.unaids.org/. Accessed November 29, 2017.

[4] UNAIDS. 90-90-90 an Ambitious Treatment Target to Help End the AIDS Epidemic. Geneva, Switzerland: UNAIDS; 2014. JC2684.

[5] UNAIDS. A Super-Fast-Track Framework for Ending AIDS in Children, Adolescents and Young Women by 2020. Geneva, Switzerland: UNAIDS; 2017 Available at: https://free.unaids.org/?utm_source=unaids-site-design&utm_medium=display&utm_campaign=en-HP_ads. Accessed November 13, 2017.

[6] Penazzato M Lule F Essajee S Paediatric HIV: the unfinished business. Lancet HIV. 2017;4:e425–e427. 28711527 10.1016/S2352-3018(17)30126-1

[7] Rudan I Gibson JL Ameratunga S Setting priorities in global child health research investments: guidelines for implementation of the CHNRI method. Croat Med J. 2008;49:720–733. 19090596 10.3325/cmj.2008.49.720 PMC2621022

[8] Rudan I Setting health research priorities using the CHNRI method: IV. Key conceptual advances. J Glob Health. 2016;6:010501. 27418959 10.7189/jogh-06-010501 PMC4938380

[9] Essajee S Bhairavabhotla R Penazzato M Scale-up of early infant HIV diagnosis and improving access to pediatric HIV care in global plan countries: past and future perspectives. J Acquir Immune Defic Syndr. 2017;75(suppl 1):S51–S58. 28398997 10.1097/QAI.0000000000001319

[10] Church K Machiyama K Todd J Identifying gaps in HIV service delivery across the diagnosis-to-treatment cascade: findings from health facility surveys in six sub-Saharan countries. J Int AIDS Soc. 2017;20:21188. 28364566 10.7448/IAS.20.1.21188 PMC5461119

[11] Ahmed S Schwarz M Flick RJ Lost opportunities to identify and treat HIV-positive patients: results from a baseline assessment of provider-initiated HIV testing and counselling (PITC) in Malawi. Trop Med Int Health. 2016;21:479–485. 26806378 10.1111/tmi.12671 PMC4881304

[12] Cohn J Whitehouse K Tuttle J Paediatric HIV testing beyond the context of prevention of mother-to-child transmission: a systematic review and meta-analysis. Lancet HIV. 2016;3:e473–81. 27658876 10.1016/S2352-3018(16)30050-9

[13] Govindasamy D Ferrand RA Wilmore SMS Uptake and yield of HIV testing and counselling among children and adolescents in sub-Saharan Africa: a systematic review. J Int AIDS Soc. 2015;18:20182. 26471265 10.7448/IAS.18.1.20182 PMC4607700

[14] World Health Organization. Information Note: Novel Point-of-Care Tools for Early Infant Diagnosis of HIV. Geneva, Switzerland: WHO; 2017 Available at: http://www.who.int/hiv/pub/toolkits/early-infant-diagnosis-hiv-2017/en/. Accessed November 29, 2017.

[15] Jani IV Meggi B Mabunda N Effect of point-of-care early infant diagnosis on retention of patients and rates of antiretroviral therapy initiation on primary health care clinics: a cluster-randomized trial in Mozambique. Presented at the International AIDS Society: AIDS2016; Durban, South Africa.

[16] Mwenda R Impact of point-of-care EID testing into the national EID program: pilot experiences from Malawi. Presented at the International AIDS Society: AIDS2016; Durban, South Africa.

[17] Technau K Sherman G Bhowan K Comparing point of care to laboratory HIV PCR testing at birth in a hospital setting in Johannesburg, South Africa [Abstract O_14]. Presented at the 8th International Workshop on HIV Pediatrics; Durban, South Africa.

[18] World Health Organization. Guidelines for Managing Advanced HIV Disease and Rapid Initiation of Antiretroviral Therapy. Geneva, Switzerland:WHO; 2017 Available at: http://www.who.int/hiv/pub/guidelines/advanced-HIV-disease/en/. Accessed November 29, 2017. 29341560

[19] Archary M Sartorius B La Russa P HIV infected children with severe acute malnutrition. Early versus delayed initiation. Conference on Retroviruses and Opportunistic Infections, Seattle, WA, 13–16 February 2017. Abstract 817LB.92.

[20] Kim MH Cox C Dave A Prompt initiation of ART with therapeutic food is associated with improved outcomes in HIV-infected Malawian children with malnutrition. J Acquir Immune Defic Syndr. 2012;59:173–176. 22107819 10.1097/QAI.0b013e3182405f8f

[21] Njuguna IN Cranmer LM Otieno VO Urgent versus post-stabilisation antiretroviral treatment in hospitalised HIV-infected children in Kenya (PUSH): a randomised controlled trial. Lancet HIV. 2018;5:e12–e22. 29150377 10.1016/S2352-3018(17)30167-4 PMC5777310

[22] ClinicalTrials.gov [Database Online]. HIV testing at family planning clinics in Mombasa county, Kenya. Identifier: NCT02994355. 2016 Available at: https://clinicaltrials.gov/show/NCT02994355. Accessed November 29, 2017.

[23] ClinicalTrials.gov [Database Online]. Active Search for pediatric HIV/AIDS (ASPA) (ASPA). Identifier: NCT03024762. 2017 Available at: https://clinicaltrials.gov/show/NCT03024762. Accessed November 29, 2017.

[24] ClinicalTrials.gov [Database Online]. Point of care virologic testing to improve outcomes of HIV-infected children. Identifier: NCT02682810. 2016 Available at: https://clinicaltrials.gov/show/NCT02682810. Accessed November 29, 2017.

[25] ClinicalTrials.gov [Database Online]. Financial incentives to increase pediatric HIV testing (FIT). Identifier: NCT03049917. Available at: https://clinicaltrials.gov/show/NCT03049917. Accessed November 29, 2017.

[26] World Health Organization. Technical Report: Combined Global Demand Forecasts for Antiretroviral Medicines and HIV Diagnostics in Low- and Middle-income Countries from 2015 to 2020. Geneva, Switzerland: WHO; 2016 Available at: http://www.who.int/hiv/pub/amds/arv-diagnostics-forecast-2015-2020/en/. Accessed December 7, 2017.

[27] World Health Organization. Meeting Report: Paediatric Antiretroviral Drug Optimization (PADO) Meeting 3. Geneva, Switzerland: WHO; 2016 Available at: http://www.who.int/hiv/pub/meetingreports/paediatric-arv-optimization-pado3/en/. Accessed November 29, 2017.

[28] Penazzato M Palladino C Sugandhi N Prioritizing the most needed formulations to accelerate paediatric antiretroviral therapy scale-up. Curr Opin HIV AIDS. 2017;12:369–376. 28570368 10.1097/COH.0000000000000378

[29] ClinicalTrials.gov [Database Online]. Prospective study of Lopinavir based ART for HIV infected children globally (LIVING Study) (LIVING). Identifier: NCT02346487. 2015 Available at: https://clinicaltrials.gov/ct2/show/NCT02346487. Accessed December 7, 2017.

[30] World Health Organization. Policy Brief: Transition to New Antiretrovirals in HIV Programmes. Geneva, Switzerland: WHO; 2017 Available at: http://www.who.int/hiv/pub/toolkits/transition-to-new-arv/en/. Accessed November 29, 2017.

[31] Clinton Health Access Initiative. ARV Market Report: The State of the Antiretroviral Drug Market in Low-and Middle-income Countries, 2015-2020. Boston, MA: CHAI; Available at: https://clintonhealthaccess.org/content/uploads/2016/10/CHAI-ARV-Market-Report-2016-.pdf. Accessed November 29, 2017.

[32] The International Maternal Pediatric Adolescent AIDS Clinical Trials (IMPAACT) Network. IMPAACT 2017: Phase I/II Study of the Safety and Pharmacokinetics of Oral and Injectable Cabotegravir and Rilpivirine in Viriologically Suppressed HIV-infected Adolescents: University of Colorado Denver NICHD CRS Available at: http://impaactnetwork.org/studies/IMPAACT2017.asp. Accessed November 29, 2017.

[33] Ford N Shubber Z Meintjes G Causes of hospital admission among people living with HIV worldwide: a systematic review and meta-analysis. Lancet HIV. 2015;2:e438–44. 26423651 10.1016/S2352-3018(15)00137-X

[34] World Health Organization. Global Tuberculosis Report. Geneva, Switzerland: WHO; 2017 Available at: http://www.who.int/tb/publications/global_report/en/. Accessed November 29, 2017.

[35] Chamla DD Essajee S Young M Integration of HIV in child survival platforms: a novel programmatic pathway towards the 90-90-90 targets. J Int AIDS Soc. 2015;18 (suppl 6):20250. 26639111 10.7448/IAS.18.7.20250 PMC4670840

[36] World Health Organization. Guideline on HIV Disclosure Counselling for Children up to 12 Years of Age. Geneva, Switzerland: WHO; 2011 Available at: http://apps.who.int/iris/bitstream/10665/44777/1/9789241502863_eng.pdf. Accessed November 29, 2017. 26158185

